# LMO2 at 25 years: a paradigm of chromosomal translocation proteins

**DOI:** 10.1098/rsob.150062

**Published:** 2015-06-24

**Authors:** Jennifer Chambers, Terence H. Rabbitts

**Affiliations:** MRC Molecular Haematology Unit, Weatherall Institute of Molecular Medicine, John Radcliffe Hospital, University of Oxford, Oxford OX3 9DS, UK

**Keywords:** LMO2, haematopoiesis, angiogenesis, chromosomal translocations, leukaemia, X chromosome-linked severe combined immuno-deficiency syndrome

## Abstract

*LMO2* was first discovered through proximity to frequently occurring chromosomal translocations in T cell acute lymphoblastic leukaemia (T-ALL). Subsequent studies on its role in tumours and in normal settings have highlighted *LMO2* as an archetypical chromosomal translocation oncogene, activated by association with antigen receptor gene loci and a paradigm for translocation gene activation in T-ALL. The normal function of LMO2 in haematopoietic cell fate and angiogenesis suggests it is a master gene regulator exerting a dysfunctional control on differentiation following chromosomal translocations. Its importance in T cell neoplasia has been further emphasized by the recurrent findings of interstitial deletions of chromosome 11 near *LMO2* and of *LMO2* as a target of retroviral insertion gene activation during gene therapy trials for X chromosome-linked severe combined immuno-deficiency syndrome, both types of event leading to similar T cell leukaemia. The discovery of LMO2 in some B cell neoplasias and in some epithelial cancers suggests a more ubiquitous function as an oncogenic protein, and that the current development of novel inhibitors will be of great value in future cancer treatment. Further, the role of LMO2 in angiogenesis and in haematopoietic stem cells (HSCs) bodes well for targeting LMO2 in angiogenic disorders and in generating autologous induced HSCs for application in various clinical indications.

## 1. The discovery of LMO2

**Figure 1.**
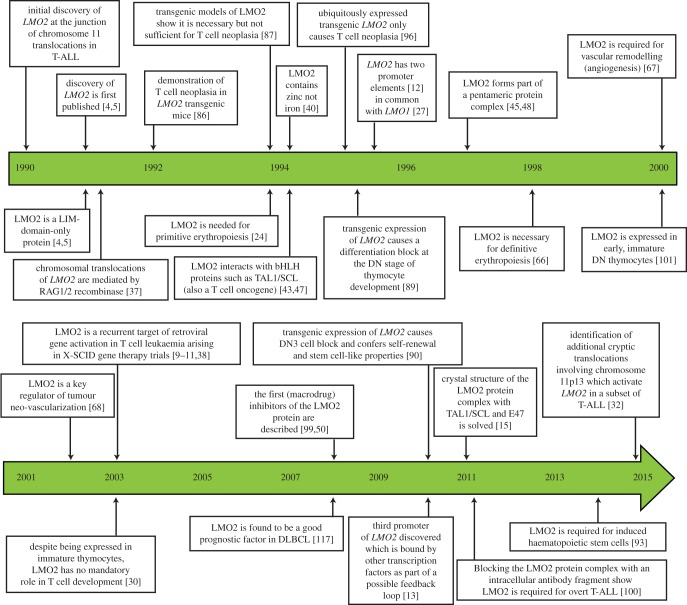
Milestones in LMO2 research: timeline indicating the major steps in LMO2 research from the gene discovery in 1990 to present.

Developments in molecular biology in the 1980s led to the finding that recurrent, cancer-associated chromosomal translocations result in either perturbed oncogene control resulting from joining with antibody or T cell receptor (TCR) genes or creation of novel fusion protein with chimaeric functions (reviewed in [1,2]). Studies of TCR gene rearrangement and gene location suggested that T cell cancer-associated chromosomal translocations would have oncogene activations [3]. This proved correct, and 25 years ago *LMO2* was discovered, and published the following year [4,5], as a recurrent chromosomal translocation partner of TCR loci in a subset of patients with T cell acute lymphoblastic leukaemia (T-ALL). Since then, research into this remarkable protein has shown that LMO2 is highly conserved among evolutionary orthologues (http://www.imm.ox.ac.uk/the-lmo-genes-and-proteins) and that it is capable of eliciting a multitude of cellular effects, ranging from a proto-oncogenic role in T cells to an essential role in haematopoiesis and vascular remodelling, as well as a major function in stem cell biology. This review outlines the developing understanding of LMO2 cancer and normal biology, illustrating how LMO2 acts as a paradigm for genes activated in acute forms of cancer. Figure 1 indicates the chronological milestones in this process.

## 2. LMO2 belongs to the LIM-domain-only family of proteins

**Figure 2.**
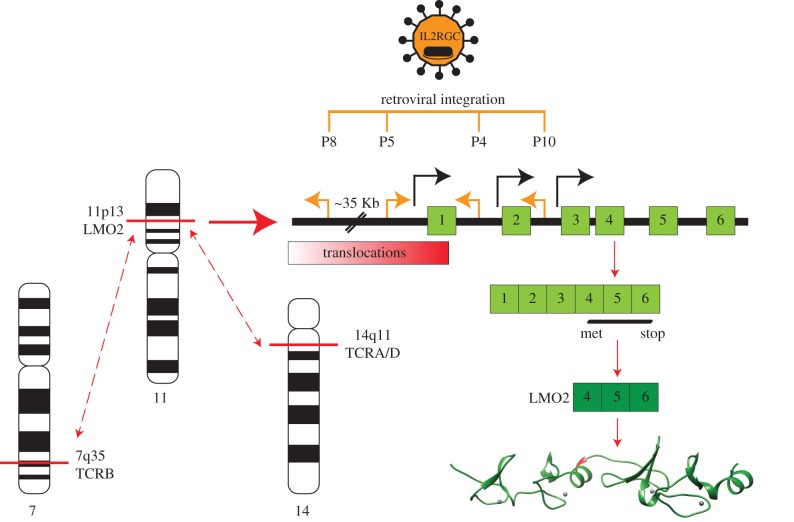
Chromosomal translocations and X-SCID retroviral insertions associated with *LMO2* gene activation. Diagram of the chromosomal bands of TCRA/D and TCRB and LMO2 involved in T cell ALL translocations resulting in *LMO2* activation. Also indicated are the retroviral insertions found in the X-SCID gene therapy trial leukaemias (orange lines, with orientation of insertion indicated by orange arrows) [9–11]. The distal, proximal [12] and intermediate [13] gene promoters are shown (black arrows). *LMO2* comprises six exons (light green boxes, numbered) of which exons 4–6 (dark green boxes, numbered) are protein coding (green ribbon structure) drawn in USCF Chimaera [14] from PDB file 2XJY [15]. The coding region of LMO2 is unaltered after either the chromosomal translocations or the retroviral insertions. (Adapted from [16].)

The progenitor gene in the family to which *LMO2* belongs was *LMO1* (formerly known as *RBTN1*, *Rhombotin-1* or *Ttg-1*). *LMO1* was one of the first T-ALL translocation proto-oncogenes to be isolated, located on chromosome 11 and involved in translocations t(11;14)(p15;q11) [6–8]. It was suggested that other similar proto-oncogenes may exist within the genome and subsequently *LMO2* (formerly known as *RBTN2*, *Rhom-2* or *Ttg-2*) was first discovered through filter hybridization experiments aimed at uncovering additional genes homologous to *LMO1* [4] and by direct cloning from the t(11;14)(p13;q11) T-ALL translocation breakpoints [5]. Thus, *LMO2*, like *LMO1*, is located on the short arm of human chromosome 11 but at band 11p13 rather than band 11p15 (*LMO1*). Leukaemias carrying translocations involving the 11p13 cluster are found more frequently in T-ALL patient samples than the 11p15 translocations [4] (figure 2).

**Figure 3.**
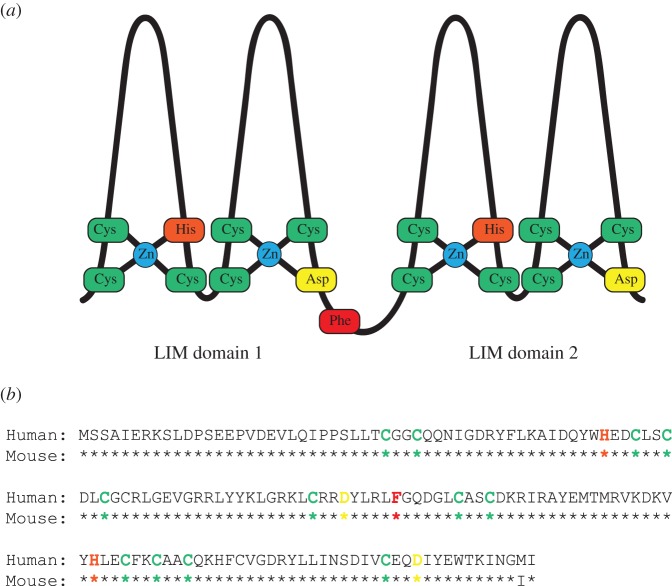
Diagrammatic structure of the **L**I**M**-**O**nly proteins and LMO2 amino acid sequence comparing human with mouse. (*a*) Schematic diagram of the LMO proteins showing the tandem arrangement of the two LIM-domains. Each domain comprises two zinc-finger-like structures, which coordinate a zinc atom between four residues. The two fingers of each domain are linked by two amino acid residues which are conserved between species and confer specificity of subsequent PPI. (*b*) An alignment of the human and mouse LMO2 proteins to illustrate species conservation and the homology and differences between them. Residues are highlighted to correlate with their position in the schematic structure shown in (*a*); green denotes cysteine, orange indicates histidine, yellow is aspartate while the key hinge region residue, phenylalanine 88, is highlighted in red [15].

The LMO family of proteins (so-called because these proteins comprise the **L**I**M-**domain-**o**nly proteins; see below) is now known to contain four genes (table 1), *LMO1*, *2*, *3* and *4* (formerly *RBTN1*, *2*, *3*, *4*) [17–20]. The name of this family comes from their tertiary structure that is composed of two tandemly arranged regions called LIM-domains (*viz*. LIM1 and LIM2). In turn, the LIM-domain was named after the transcription factors **L**in-11, **I**sl-1 and **M**ec-3 [21–23]. It is a cysteine-rich motif, characterized by the consensus amino acid sequence C-X_2_-C-X_17−19_-H-C-X_2_-C-X_2_-C-X_16−20_-C-X_2_-C-X/C/D (figure 3).

**Table 1.** Chromosomal location of the *LMO* gene family in human and mouse genomes. Chromosomal translocations known to occur in proximity to the genes are listed, with associated malignancies and knock-out mouse phenotypic defects indicated. For references, see detail in relevant text sub-sections.

	chromosome band					
protein	human	mouse	knock-out phenotypic defects	chromosomal translocation	T-ALL	other cancers
LMO1	11p15	7E3	CNS	t(11;14)(p15;q11)	✓	neuroblastoma
LMO2	11p13	2E2	haematopoiesis	t(11;14)(p13;q11) t(7;11)(q35;p13)	✓	B cell lymphoma B-ALL
LMO3	12p12	6G1	CNS	t(7;12)(q35;p12)	✓	neuroblastoma
LMO4	1p22.3	3H2	neural tube development	not known	not known	breast cancer neuroblastoma

LMO1 and LMO3 share the highest degree of sequence conservation, being 98% identical. *LMO1*, *2* and *3* are all involved in T-ALL by chromosomal translocations (table 1), but *LMO4* (uncovered through two hybrid screening [18,20]) is the most divergent member of the family and currently has no known leukaemia translocations. Gene targeting has been implemented to discover the gene functions in mice. The homozygous phenotypes of the four genes in knock-out mice show their essential role in cell fate decisions (table 1), and there is phenotypic synergy when homozygous loss of *Lmo1* and *Lmo3* occurs in mice [24,25]. These phenotypic properties in part led to the proposal of the ‘master gene’ hypothesis [1] based on highly conserved, developmentally important, transcriptional activators [1,26]. LMO2 is an archetypal example of such a master transcription regulator, but also of a chromosomal translocation oncogene, which is discussed further in the following sections.

## 3. Chromosomal translocations of LMO2 and the involvement of RAG recombinase

Human LMO2 has six exons, of which the last three encode the protein comprising 158 amino acids and has two major transcription promoters [12,27,28] and a recently described third ‘intermediate’ promoter [13] (indicated in figure 2). Gene expression analyses have shown LMO2 is expressed in a range of tissues during development [28,29] but not in normal mature thymocytes other than tumourigenic T cells [5,17,24,30]. This suggests that LMO2 expression in T cells is reliant upon activation through mechanisms such as chromosomal translocations.

Chromosomal aberrations, including translocations, deletions and insertions are frequent in childhood T-ALL, often resulting in the temporally and spatially incorrect activation and expression of developmental regulatory genes [2]. Aberrant expression of LMO2 resulting from such gross genetic abnormalities is documented in approximately 9% of childhood T-ALL cases [31,32] but expression has been reported in as much as 45% of T-ALL [33].

**Figure 4.**
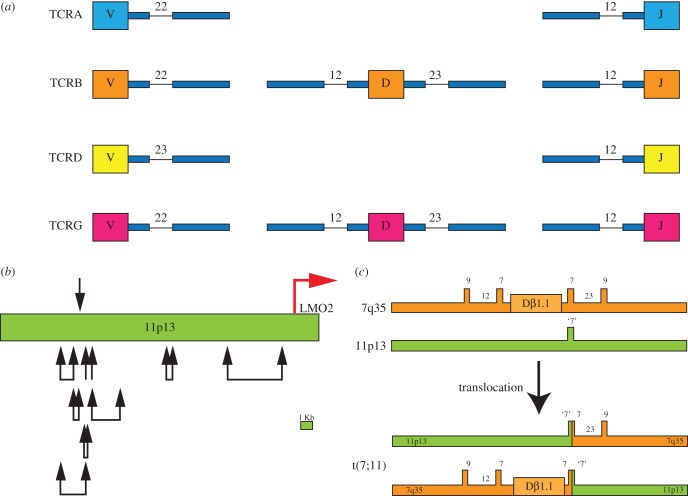
Site-specific mechanism of *LMO2* chromosomal translocations mediated by RAG recombinase. (*a*) TCR gene segments (V, D and J) are linked to RSS. Each segment has heptamer and nonamer sequences as indicated (and at the spacings in TCR V, D and J segments shown). (*b*) Heptamer sequences are found near LMO2 at chromosome 11p13 (green), specifically around translocation breakpoint regions denoted by black arrows [37]. Although not all were found to possess joining via the heptamer sequences, hallmark sequences of Rag recombinase were noted. The location of the proximal LMO2 promoter is shown by a red arrow. (*c*) Detailed analysis of the reciprocal translocation breakpoints of LMO2 has shown the involvement of the VDJ RAG recombinase in the translocations. An example of the organization of reciprocal t(7;11)(q35;p13) translocation regions is shown (lower part of the panel) compared with germline organization. Note the presence of a heptamer-like sequence at the junction of the 11p13 breakpoint [37].

*LMO2* occurs at the junction of common T-ALL-associated translocations, namely the translocation t(11;14)(p13;q11) involving the *TCRD/A* from 14q11 or the t(7;11)(q35;p13) translocation with the *TCRB* locus from 7q35 (figure 2). These translocations encompass approximately 5% of primary paediatric T-ALL cases investigated by karyotypic analysis [32]. The mediator of these translocations, and of other similar leukaemic TCR-locus translocations such as involving *TAL1/SCL*, herein referred to as *TAL1*, [34,35], appears to be the RAG1/2 recombinase complex that is normally responsible for intra-chromosomal antigen receptor gene rearrangement in thymic T cell maturation (reviewed in [36]), but that makes occasional errors of inter-chromosomal translocation [37]. In many cases, this error is sequence-specific as the chromosomal translocation breakpoint sequences on chromosome 11p13 (i.e. near *LMO2*) can have heptamers typical of recombination signal sequences (RSS) that could be the normal recognition for RAG recombinase, albeit generally lacking the nonamer stretch found at genuine RSS locations. Figure 4*a* illustrates normal TCR heptamer and nonamer RSS for variable (V), diversity (D) and joining (J) for *TCRD* and *TCRB*, and V-J RSS for *TCRA* and *TCDG*. When the breakpoint cluster region near *LMO2* on chromosome 11 was sequenced, heptamer-like RSS were found near translocation breakpoints (figure 4*b*) and in three independent tumours, the break occurs at the same heptamer-like RSS on chromosome 11 [37].

The precision with which the chromosomal translocations subvert TCR rearrangement is illustrated in figure 4*c*, which shows the molecular architecture of the one t(7;11) case [37]. Although the TCR-associated translocations are sequence-specific, their rarity doubtless reflects a restriction imposed by chromosomal territory in the T cell nuclei and less efficient processing when heptamer, but not nonamer, RSS are present.

*LMO2* expression may also be activated by the cryptic deletion, del(11)(p12p13) in approximately 4% paediatric T-ALL patients [31], and by a plethora of cryptic and newly discovered translocations [32]. It is intriguing that the del(11) can involve the juxtaposition of *RAG2* with *LMO2* (and, apparently, deletion of *RAG1*). The deleted region, just upstream of *LMO2*, includes negative regulatory sequences, whose removal could permit *LMO2* expression via its proximal promoter. These same sequences are also removed from *LMO2* by the chromosomal translocations. However, the existence of translocation-negative, LMO2-positive clonal T cell tumours in up to 45% of other T-ALL [33] suggests that *LMO2* may be activated through mechanisms other than gross genetic changes at the locus. This may arise through TAL1, LYL1 and/or ERG cooperatively engaging in a feedback loop at their respective regulatory elements to activate or increase expression [13] (see §5).

## 4. *LMO2* is a preferred oncogene in X chromosome-linked severe combined immuno-deficiency syndrome gene therapy associated leukaemia

Aberrant expression of LMO2, following activation by retroviral insertion, has also led to a T-ALL-like leukaemia arising in four of the children treated in X chromosome-linked severe combined immuno-deficiency syndrome (X-SCID) gene therapy trials [9,10,38]. X-SCID is caused by a defect in the Interleukin-2 receptor gamma common chain (IL2RGC), which is necessary for high-affinity signalling of several interleukin (IL) receptors and is typified by a lack of mature T cells and natural killer cells. Gene correction was achieved by transduction of autologous CD34^+^ bone marrow stem cells with retrovirus expressing *IL2RGC* [39]. While several patients eventually had an immune cell repertoire that matched those of non-SCID children, some of the children involved in the trial developed T cell leukaemia [10,11,38,39]. Four cases have activated LMO2 caused by retroviral insertion into, or just upstream of, the *LMO2* locus that consequently activates expression of LMO2 through the strong constitutive expression of the retroviral promoter/enhancer systems. Figure 2 shows the location of the insertion sites and that insertional mutagenesis occurs outside the *LMO2* coding region in all four cases. We have shown with a mouse transgenic model that concurrent expression of *LMO2* and *IL2RGC* in thymocytes accelerates the formation of clonal T cell neoplasias compared with those arising in transgenic aberrantly expressing only *LMO2*, while aberrant expression of *IL2RGC alone* has no discernable oncogenic effect in thymocytes (K. Ruggero & THR, unpublished data). This demonstrates that *LMO2* and *IL2RGC* expression synergizes in thymus cells to cause T cell neoplasias in mice and, by inference, describes the T cell adverse effects that occur in the X-SCID gene therapy patients. A model for LMO2-associated X-SCID T-ALL is discussed in §9.

## 5. LMO2 protein structure and the multimeric DNA-binding complex

LMO2 is a LIM-domain-only protein. LIM-domains were originally thought to carry Fe-S centres but the demonstration of zinc in the proteins [40] led to the discovery that LIM-domain folds to form two LIM-fingers through the coordination of a zinc atom by the side-chains of three cysteine residues and a histidine residue (finger 1), or three cysteine residues and an aspartic acid residue (finger 2) (figure 3). The intervening peptide chain is forced outward to form the finger-like projections, arranged as two perpendicular anti-parallel β-sheets [41]. The LIM-fingers are distinct from the zinc-fingers of transcription factors such as those of the GATA-family, as no evidence exists of LIM-domain-only proteins directly binding DNA. Rather, the LIM-fingers provide a mechanism of protein : protein interaction (PPI) with a variety of binding partners [42–45]. Each finger is separated by two amino acid residues, the spacing and sequence of which has been shown to be vital for subsequent protein interactions in other LIM-domain-containing proteins [46].

**Figure 5.**
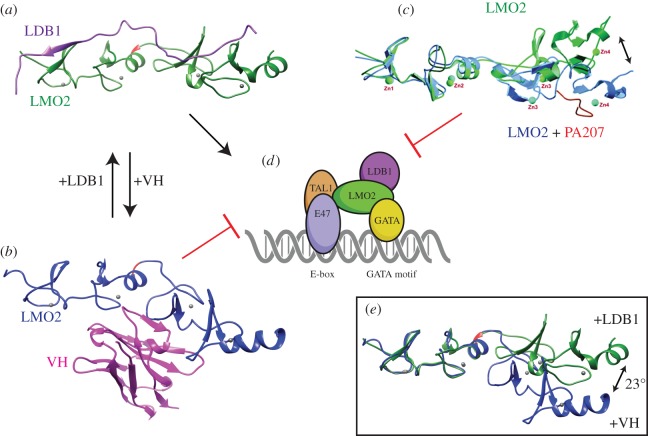
Representation of the conformational flexibility of LMO2 in complex with LDB1 or inhibitors of LMO2. The pentameric LMO2-complex commonly found in erythroid lineage cells binds to a bipartite E-box-GATA motif [45] and is permitted or inhibited depending upon the stabilizing protein partner of LMO2 (*a*,*d*). LMO2 has been shown to nucleate variants of this complex, including the substitution of the GATA-factor and binding other bHLH transcription factor proteins [47], with resulting altered DNA recognition sequence binding specificity [48]. The structure and conformational stabilization of LMO2 (green) when bound with LDB1-LID (purple) [15] is indicated in (*a*). When LMO2 (shown in blue in panel (*b*)) is in complex with the anti-LMO2 VH (magenta), there is a conformational distortion, preventing nucleation of the LMO2-complex to the complex illustrated in (*d*) [49]. Superimposition of the normal LDB1- and VH-bound structures highlights the 23° contortion of the molecule when the anti-LMO2 VH is bound (*e*). The hinge region residue Phe88 (red), zinc atoms coordinated by the LIM-fingers (grey spheres) are indicated in both structures, drawn using UCSF Chimaera [14] from PDB accession codes 2XJY [15] and 4KFZ [49]. An *in silico* model of the structural effects on LMO2 induced by the peptide aptamer PA-207 is shown in (*c*). (Adapted from [50].)

LMO2 does not bind DNA directly but instead acts by forming part of a bridge for DNA-binding proteins to create a bipartite multi-protein DNA-binding complex (illustrated in figure 5*d*). Newly synthesized LMO2 protein does not have a stable conformation, requiring interaction with other proteins to stabilize its inherently unstructured nature and also prevent proteasomal degradation, as in the case of TAL1 binding [51]. LMO2 was first shown in complex with TAL1, E47 and GATA-1 in erythroid cells [43], and subsequently shown to interact with several other basic-helix-loop-helix (bHLH) transcription factor proteins including LYL1 and TAL2 [47]. LMO2 binds directly to the bHLH protein TAL1 and it is intriguing that this partner is also a T cell oncogene [34,35,52–54]. TAL1 is a haematopoietic tissue-specific member of the class II bHLH transcription factor family, which also includes LYL1 and TAL2, both of which interact with LMO2 [47]. LMO2 can bind GATA-1, GATA-2 or GATA-3 [45,55,56]. The LMO2-complex contains an additional protein called the LIM-domain-binding protein 1 (LDB1) [45,57]. The LMO2-complex thus comprises the bipartite DNA-binding complex recognizing an E-box and GATA site, separated by one turn of the DNA helix (figure 5*d*). This complex was shown capable of substituting the GATA-factor for another TAL1/E47 heterodimer in transgenic *LMO2-*dependent T cell tumours [48] and also at the *Ckit* promoter in erythroid cells [58]. Similarly, a GATA-E-box-GATA motif has been shown at the erythroid Krüppel-like factor locus [59]. Taken together, these findings suggest that LMO2 has a flexible multi-protein complex nucleation function in different cell settings.

As it is a naturally disordered protein, containing very little secondary structure, LMO2 presents an unstable conformation, making it inherently insoluble when made in recombinant form, and hence difficult to purify for structural studies. This has been partially overcome by expressing both LIM-domains individually or full-length LMO2 as a fusion protein with the LIM-interaction domain (LID) of LDB1 [15,60,61]. NMR [60,62] and crystallographic data [15,63] of the LMO2–LID fusion show that LID binds across the LIM1 and LIM2 domains. There is a flexible hinge region located between the two LIM-domains and the LDB1 LID interaction, leaving the LIM-finger-side of both domains unoccupied for other protein interactions [15]. Thus, the structural instability of LMO2 is resolved on binding LDB1 (as illustrated in figure 5*a*), allowing the nucleation of the additional members into a multimeric protein complex, such as the erythroid complex including TAL1/E47 and GATA1 binding to the bipartite E-box-GATA motif [45]. When bound to the N-terminal LIM-domain of LMO2, the TAL1/E47 heterodimer undergoes small conformational changes [64] that may be a feature allowing fine-tuning of the LMO2-complex to repress or activate specific genes in a temporally controlled fashion. Conformational change in LMO2 has been induced by binding to an antibody fragment [49] that accounts for an inhibitory effect on the LMO2 protein complex (figure 5*b*,*e*) (discussed further in §10).

## 6. LMO2 haematopoietic master regulator function portends its oncogenicity

After the identification of *LMO2* as a proto-oncogene in T-ALL, the natural function of the protein was investigated in mouse and human cells to delineate its normal biochemistry and to instruct future work on development of anti-LMO2 therapies. A principle finding was the expression of LMO2 in haematopoietic cells and a pivotal function in specification of haematopoietic lineage. This suggested the means by which LMO2 function lies behind its role as a cancer inducer.

**Figure 6.**
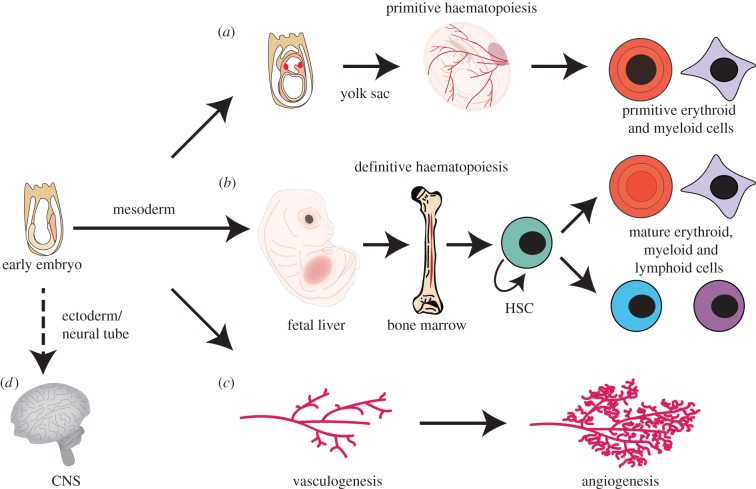
Role of LMO2 in mouse development. Gene knock-out of mouse LMO2 has revealed its involvement in several aspects of haemaotopoiesis and blood vessel formation. LMO2 has a necessary role in primitive [24] (*a*) and definitive haematopoiesis [66] (*b*), and it is required for remodelling of the vascular networks as part of angiogenesis [67] (*c*). LMO2 has been shown to be expressed in the developing central nervous system [17] and tissue staining using lacZ knock-in mice showed specific regions of LMO2 expression [66] although no discernable knock-out brain phenotype has been noted to date.

The development of gene targeting in mouse embryonic stem cells (ES) and generation of mice from these mutant pluripotent cells (technology reviewed in [65]) allowed LMO2 gene targeting to be implemented to discover the functions of LMO2 in mouse development [24,66]. Insertion of the β-galactosidase gene as reporter for *Lmo2* expression showed mainly expression in haematopoiesis and in developing brain [67,68]. Figure 6 outlines LMO2 functions in development, determined mainly by gene targeting. In the haematopoietic hierarchy, there is an essential role for LMO2 in red cell development in the embryo yolk sac (primitive erythropoiesis) (figure 6*a*) as homozygous *Lmo2* null mice died *in utero* at embryonic stage E9–E10 [24]. While *Lmo2* null embryos displayed normal blood islands of the yolk sac, they were devoid of red cells. Intriguingly, it was later shown that the consequences of the homozygous *Lmo2* null mutation was similar to those encountered in *Tal1* [69,70], *Gata-1* [71,72] and *Gata-2*-null embryos [73], presaging the finding that the LMO2 protein is part of a pentameric complex that includes TAL1 and GATA proteins [45,47,55].

As *Lmo2* null embryos do not survive to term, a role of LMO2 in adult haematopoiesis (definitive haematopoiesis) required a different strategy for analysis. Definitive haematopoiesis initiates in the mid-gestation embryo in the aorta–gonad–mesonephros region (reviewed in [74,75]), switches to the fetal liver and relocates to the bone marrow shortly before birth, where it persists throughout adult life. Accordingly, ES cells with homozygous *Lmo2* null mutation were used to create chimaeric mice that have contribution of cells from both the recipient blastocyst and the donor homozygous *Lmo2* null ES cells. These studies showed that homozygous *Lmo2* null ES donor cells do not contribute to haematopoiesis in adult mice [24,66], suggesting that LMO2 function is crucial in the multi-potent haematopoietic stem cells (HSCs) (figure 6*b*). It was also shown that overexpression of LMO2 (and LDB1) in erythroblast cell lines resulted in immature, undifferentiated precursor erythroid cells, which established that LMO2 could act as a negative regulator of differentiation [76]. Furthermore, LMO2 mRNA expression has been demonstrated during both primitive [77] and definitive [78] haematopoiesis through *in situ* hybridization of mouse embryos.

Blood vessel endothelial cell formation (de novo vasculogenesis) also arises from mesoderm and vascular remodelling (angiogenesis) is growth of new blood vessels from existing vasculature through sprouting, migration and adhesion. The processes of blood cell and blood vessel specification are tightly linked (reviewed in [79]). By examining the contribution of *Lmo2* null-LacZ ES cells in chimaeric mice, it was found that Lmo2 is not required for the de novo vessel formation during early embryogenesis but is needed for angiogenic remodelling of vascular networks (figure 6*c*) [67].

LMO2 also has a role in normal endothelial and lymphatic endothelial cells. It is found in lymphatic endothelial cells, which form new lymphatic vessels by lymphangiogenesis, a process similar to angiogenesis. Angiogenesis can be induced by various growth factors, including VEGF that increases endothelial cell and lymphatic endothelial cell levels of LMO2 and GATA2 [80]. Moreover, through an *in silico* screen [81], the E-box-GATA motif of the LMO2-transcriptional complex was found to occur in the promoter regions of many pro- and anti-angiogenic-related genes such as *Angiopoietin-2*.

LMO2 has long been implicated in developing CNS tissue (figure 6*d*) [17] and is expressed in the adult mouse brain, alongside LMO1 and LMO3 [82], particularly after epileptic seizures, possibly as a repair mechanism. Intriguingly, LMO2 can nucleate an altered transcriptional complex with LDB1, the bHLH E-box-binding transcription factor NSLC2 and BEX2 in fetal brain tissue [83] to regulate transcription. There remains no clear correlation between this CNS expression of LMO2 and function but it may be a link to the recent implication that LMO2 plays a role in glioma [84] (see §11).

## 7. LMO2, haemtopoietic stem cells, iPS and cancer-initiating cells

LMO2 has a role in stem cells and/or confers stem cell-like functions. As discussed in §6, LMO2 is needed in induction and maintenance of HSCs that can differentiate into all blood cell types (HSCs are described as pluripotent in the context of blood cell development to distinguish them from multi-potent progenitors). The gene is expressed in mouse lineage-negative, ScaI-positive, cKit-positive (LSK) haematopoietic progenitor cells in mice [85], and knock-out of *Lmo2* prevents primitive and definitive haematopoiesis [24,66] (see also §6).

**Figure 7.**
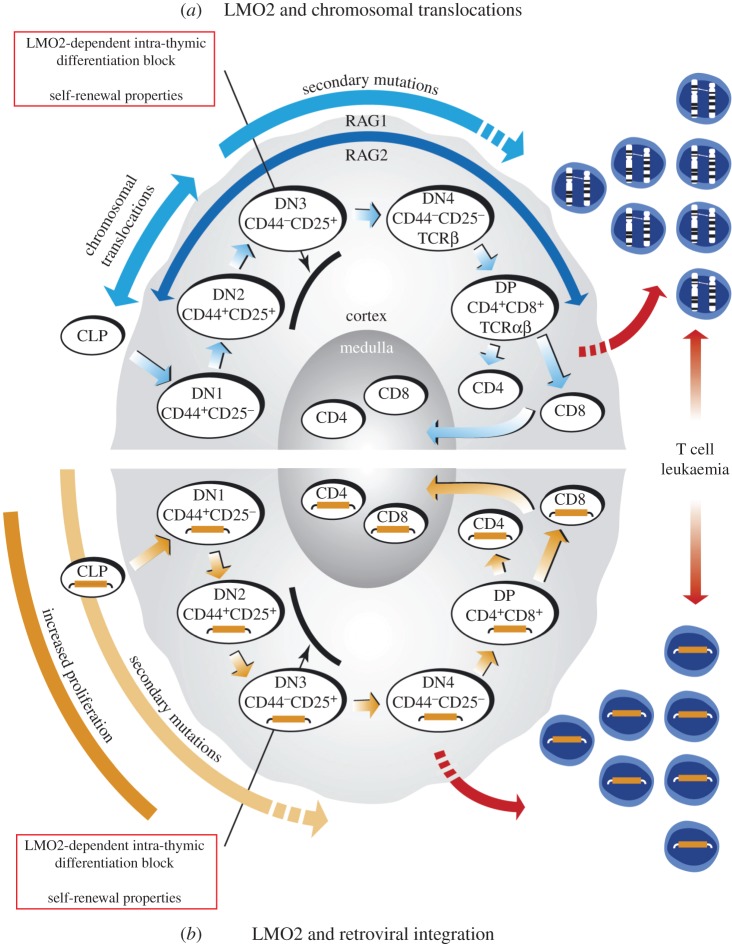
Model of LMO2-mediated leukaemogenesis. Schematic diagram to represent the current understanding of the effect of LMO2 in LMO2-translocation positive T-ALL (*a*) and in the X-SCID gene therapy trial leukaemias (*b*) where LMO2 expression was activated through retroviral integration. (*a*) Fully mature CD4 or CD8 single positive (SP) T cells develop in the thymus through DN CD4/CD8 precursor populations, DN1–4, which are characterized by their progressive changing expression of CD25 and CD44 surface markers. The RAG recombinase genes are expressed from the DN2 stage onwards and promote TCR gene recombination events to produce functioning receptor molecules during the normal maturation cycle of the early thymoctyes. Although some cells progress through to mature CD4/CD8 double positive or SP T cells, thymus-specific transgenic *Lmo2* mice exhibit a block in T cell differentiation with an accumulation of DN2–DN3 stage immature thymocytes (indicated), which after a long asymptomatic phase develop clonal T cell neoplasias [87–89] after the acquisition of additional mutations [53]. This differentiation block coincides with the timing of RAG expression, directly mimicking the temporal occurrence, and effect of, chromosomal translocations involving the *TCR* genes and *LMO2* loci [37]. Furthermore, the enforced expression of *Lmo2* in the transgenics results in immature cells with acquiring self-renewal capacity, demonstrated by serial transplantation of the DN3 thymocytes [90]. (*b*) Following viral therapy in the X-SCID gene replacement trials, leukaemias were observed in which retroviral insertion had occurred into or upstream of the *LMO2* gene and activated expression in thymocytes [9–11]. Expanding from the LMO2 tumourigenesis model in (*a*), it would appear that leukaemogenesis in the X-SCID patients could arise from lymphoid progenitor cells with activated *LMO2* expression migrating to the thymus, as part of the normal maturation process, and resulting in LMO2 conferring the DN2/3 differentiation block that was observed in the transgenic model, promoting self-renewal properties, accumulation of secondary mutations and finally the severe clinical adverse effects of leukaemias seen. (Adapted from [16].)

One of the key functions of LMO2 in T cell neoplasia is interference with intra-thymic T cell differentiation as the immature double negative (DN; not expressing CD4 or CD8) thymocytes accumulate in *Lmo2* transgenic mice [86,87] (illustrated in figure 7*a*). This is in part achieved in DN3 cells, where LMO2 elicits self-renewing properties to these cancer precursors [90]. A critical function of LMO2 in T cell tumourigenesis is thus to form leukaemia-initiating cells (LICs) as a pool for further oncogenic mutations (discussed in §9).

An exciting consequence of LMO2 stem-cell properties is the recent demonstration of LMO2 as a factor in the generation of induced haematopoietic stem cells (iHSCs). This follows from work demonstrating the creation of pluripotent stem cells (iPSCs) through reprogramming of terminally differentiated adult cells by the expression of key transcription factors [91]. Three studies have defined the requirement for LMO2 together with other factors to support creation of stem cell properties in differentiated cells, to generate induced haematopoietic cells. One study shows that expandable haemangioblasts (capable of forming endothelial cells, multi-lineage haematopoietic cells and smooth muscle) can be made *in vitro* from ES cells, fetal liver cells and fibroblasts [92]. LMO2 with its partners TAL1 and GATA2 together with SOX17, PITX2 and MYCN are sufficient. iHSCs capable of multi-lineage development can be formed from fibroblasts by expressing LMO2 with its partners TAL1 and GATA2 together with ERG, RUNX1 [93], and it is possible to reprogram committed cells (myeloid and lymphoid committed cells) using a cocktail of factors in addition to LMO2 including RUN1T1, HLF, PRDM5, PBX1 and ZFP37 [94]. In the latter, it is possible that the LMO2 partners (GATA and TAL1) are provided by the cellular environment in the committed cells used for making the iHSC. Autologous iHSCs that are capable of multi-lineage differentiation and of long-term reconstitution have enormous potential clinical benefit. There may be a risk involving the oncogenic potential of LMO2 in reprogrammed cells [95], particularly given the appearance of leukaemias in the X-SCID trials following retroviral insertional activation of *LMO2* [9,10,38] and the evidence of neoplasia in models of enforced *lmo2* expression [87–89,96]. Stringent controls and safety measures may be necessary for further evaluation of these technologies, possibly through using exogenous protein rather than genetic manipulation, to avoid these problems [97].

## 8. LMO2 and the chromosomal translocation master gene model

The chromosomal translocation master gene model [1] was proposed based on the normal role of the genes found at the junctions of acute leukaemia chromosomal translocations, such as the two LIM-only genes *LMO1* and *LMO2*, and *HOX11*. In particular, LMO1 was shown to have specific expression during hindbrain rhombomere development [98]. The essential features of the model were based on the findings that these genes play a role in lineage determination and/or tissue specification and the suggestion that the molecular mechanisms underlying these functions are subverted, after the chromosomal translocation gene activation, to create the ‘chromosomal translocation master gene’. The proteins are involved in PPI and DNA binding (indirectly like LMO2 or directly like TAL1 or HOX11) and thus control gene expression. Therefore, the cell that gains a chromosomal translocation (or other chromosome abnormality leading to aberrant gene expression) is committed to a transcriptional programme that the chromosomal translocation master gene dictates.

The subsequent work on LMO2 shows that it fulfils all the components of the master gene model; (i) the protein is active in controlling PPI by forming multimeric protein complexes that bind to DNA through two parts of the complex (such as bHLH and GATA components), (ii) it confers a differentiation block on T cell development (intra-thymic), and (iii) it also confers the stem-cell-like property of self-propagation on these cells. Inhibition of the LMO2 protein complex in mouse models of T cell neoplasia [50,99,100] show that LMO2 is necessary, but not sufficient [87], for the cancers, but that it is required at the stage of overt disease.

## 9. The *LMO2* paradigm of chromosomal translocation genes in T cell acute lymphoblastic leukaemia

The combined studies of transgenic T cell neoplasias resulting from thymic expression of *Lmo2* and the study of human T-ALL carrying *LMO2* translocations led to a model of LMO2-mediated T-ALL (figure 7*a*) that is a paradigm for chromosomal translocation-associated acute cancers.

Transgenic expression of *LMO2* from *CD2* [87–89], *Metallothionine* [96] or *Lck* promoters (K. Ruggero & THR, unpublished data) results in long-latency, clonal T cell neoplasia. While not expressed in normal mature T cells, gene expression analyses have shown that LMO2 occurs in early, immature CD4/CD8 DN thymocytes (thymocytes that do not express TCRs), at the DN1 stage of their development alongside TAL1 [101], a member of the LMO2 multi-protein transcription factor complex. Normally, downregulation of LMO2 and TAL1 is concordant with upregulation of additional transcription factors that permit thymocyte maturation through the DN3, DN4 and double-positive CD4/CD8 stages, resulting in fully mature single CD4-positive or CD8-positive T cells. Despite evidence of LMO2 being expressed in the DN1 early thymocytes, lymphoid lineage-specific *Lmo2* knock-out showed no abnormal development or irregular thymic cellularity [30].

The detailed study of tumourigenesis caused by transgenic *LMO2* from the *CD2* [87–89] or from the *Lck* promoter (K. Ruggero & THR, unpublished data) demonstrates that LMO2 exerts its oncogenic effect on immature T cells in the thymus, prior to TCR-based positive and negative selection (figure 7). The transgenic mice exhibit two phases in the appearance of overt T cell neoplasias, the first being a long asymptomatic phase during which there is an accumulation of immature thymocytes within the thymus [53,87,89] at the DN2/DN3 stages (defined, respectively, by CD25^+^/CD44^+^ or CD25^+^/CD44^−^). Later, overt T cell neoplasias develop with clonal TCR rearrangements. Thus, transgenic *LMO2* is necessary in this model but not sufficient, and transition to frank haematopoietic cancer requires secondary mutations. One type of mutation that is found frequently in the transgenic mouse tumours are *Notch1* mutations (K. Ruggero & THR, unpublished data), which parallels mutations seen in about 50% of human T-ALL [102].

The data suggest that, in transgenic models, T cell progenitors enter the thymus as DN1 cells (CD25^−^/CD44^−^) and begin to mature through DN2 and DN3 but are held up at the DN3 stage (figure 7). This is a stochastic block as some cells progress to make mature T cells expressing CD4^+^/CD8^+^ intermediates or CD4^+^ or CD8^+^ T cells. Serial transplantation studies show that the DN3 thymocytes of the *CD2–LMO2* transgenic mice acquire stem cell-like properties as these cells have self-renewal properties in recipient mice [90]. We propose that this sub-population of self-renewing DN3 cells acts as a pool of LICs. The high penetrance of transgenic overt T cell neoplasia is a consequence of there being a pool of LICs in which secondary mutational events can arise. Of course, in the case of the human *LMO2* chromosomal translocation T-ALL, cancers arise from single cells due to the rarity of the relevant translocation. Interestingly, transgenic mice expressing LMO2 with its partner protein TAL1 exhibit clonal T cell neoplasias at a faster rate than in *LMO2*-only transgenics [53,87], whereas *TAL1*-only mice did not show any neoplasias [103]. This latter finding may reflect the lack of endogenous LMO2 in the critical thymocyte (i.e. DN3) to partner the TAL1 transgenic protein, whereas the LMO2 transgenic protein can partner other endogenous bHLH proteins (e.g. LYL1) in the absence of TAL1 for tumourigenesis.

We discussed in §3 how the *LMO2* chromosomal translocations are frequently mediated through mistakes made by RAG1/2 recombinase causing the inter-chromosomal events. As RAG1 and RAG2 begin expression at the early stages of the DN thymocyte differentiation, and are active in DN2/DN3 cells, it is likely that *LMO2* chromosomal translocations occur before the DN3 stage and have a pathogenic consequence as the single intra-thymic translocation event will result in a LIC. By analogy with the transgenic mouse data, this cell will acquire self-renewal properties due to LMO2 expression and propagate to gradually produce a pool of cells within which the secondary mutations (such as *NOTCH1*) occur to give rise to frank leukaemia (figure 7*a*). An interesting possibility is that RAG-mediated translocations could occur throughout thymocyte differentiation but only manifest tumourigenic consequences if before or at the DN3 stage. In addition, it seems significant that LMO2 translocations do not appear in B cell neoplasias, despite B cells having RAG1 and RAG2 expression.

This chromosomal translocation model provides an intriguing back-drop to the incidence of *LMO2*-associated gene activation in the X-SCID gene therapy trials. Parallels with the effect of *LMO2* chromosomal translocations are remarkable (figure 7*b*; reviewed in [16,104,105]). Autologous donor CD34^+^ bone marrow HSCs from X-SCID patients were infected with retrovirus expressing *IL2RGC* and in some recipients leukaemias developed with LMO2 activation. In the model (illustrated in figure 7*b*), it is envisaged that accessibility for retroviral insertion into *LMO2* in the pluripotent CD34^+^ bone marrow cells occurs as LMO2 is expressed therein and thus the chromatin is ‘open’. But it is also predicted that this has no oncogenic consequence until the progenitors enter the thymus as all models show that LMO2 is only oncogenic during haematopoiesis in thymocytes. The cell(s) with the retrovirally activated *LMO2*, by analogy to chromosomal translocation-activated *LMO2*, can progress normally into the DN3 stage, where the presence of LMO2 will cause inhibition of T cell differentiation and the LIC phenotype. This clone will eventually acquire secondary mutation(s) that in turn lead to overt leukaemia. The cooperating property of the LIC(s) in the gene therapy patients, which influences the adverse outcome, is that the *IL2RGC* gene is the common chain of several IL receptors, including the IL2 receptor, which is expressed in DN3 cells (marked by CD25 surface protein which is IL2RA).

Recent clinical studies have identified a highly aggressive form of T-ALL, designated early T cell precursor-like ALL (ETP-ALL) [106], which is characterized by immature DN1-like immature thymocytes and which is often highly treatment-resistant. ETP-ALLs have been found to have high levels of LMO2 and LYL1 expression [107–109], and LYL1 appears to be the necessary bHLH transcription factor partner of LMO2 rather than TAL1 [108]. Together, the LMO2–LYL1 complex seems to correlate with enhanced rates of transition to full neoplasia, perhaps an effect from withdrawal of competitor cells within the early DN populations, promoting existing thymocyte self-renewal and thus the acquisition of further mutations [110].

## 10. Pre-clinical macrodrugs targeting the LMO2 protein complex

The transgenic models of LMO2-associated T cell neoplasia show that LMO2 is necessary but not sufficient for T cell neoplasia. Because of this, it is possible that LMO2 is only needed in the asymptomatic phase within the LIC and does not need to persist in overt tumours. Such a hit and run mechanism has been ruled out in the transgenic tumours by inhibiting the LMO2 protein with intracellular macrodrugs (*macro*molecular *drugs*) exemplified by a peptide aptamer [50], by an antibody single-chain Fv fragment [99] and by a single VH domain antibody fragment [49,100].

Each of these macrodrugs binds to LMO2 and interferes with LMO2 PPI by different mechanisms. The peptide aptamer confers a growth inhibition on LMO2 leukaemic cells in transplantation assays [50], and modelling suggested that it disrupts the LMO2 LIM finger 4 by competing for interaction with the zinc atom and thus destroying the ability of LMO2 to interact with components of the multimeric complex (figure 5*c*). While recent data imply broad binding of the aptamer across other zinc-finger-containing proteins [111], *in vivo* binding shows specificity for LMO2 and mutagenesis suggests predominance of binding to LIM finger 4 [50]. The intracellular antibody fragments also abrogate the neoplastic effect of LMO2 in transgenic *LMO2* cell transplantation assays [99,100] mediated by the disruption of the LMO2-complex by preventing LDB1 interaction [100]. The crystal structure of the LMO2 : VH complex [49], compared with that of LMO2-LDB1-LID fusion [15], demonstrates the mechanism. When the VH binds to LMO2, the latter adopts a distorted conformation around its hinge region (figure 5*b*,*e*). This bending and twisting distortion of a normal LMO2 fold induced by the natural partner LDB1 LID, interferes with the interaction with natural partners of LMO2. Thus, the intracellular single-domain antibody fragment makes its binding partners unavailable for the normal protein complex formation and function.

## 11. Association of LMO2 with non-T cell tumours

The chromosomal abnormalities involving *LMO2* appear to be restricted to T cell tumours, but LMO2 expression studies have implicated LMO2 in a range of other cancers. It is noteworthy nevertheless that widespread transgenic expression of LMO2 from a *Metallothionine* promoter only resulted in T cell tumour phenotype [96], implying that the tumourigenic effect in mice is restricted to the T cell lineage.

LMO2 expression has been observed in diffuse large B cell lymphoma (DLBCL) [112] and in some B cell acute leukaemias [113–115]. It is expressed in normal germinal centre (GC) B cells and in the GC-derived DLBCL [112,116] where the presence of LMO2 is a good prognostic factor [117]. This may reflect that LMO2 expression is a passenger effect of pre-existing expression in GC cells or it may be a consequence of there being different LMO2-binding partners in DLBCL, where it has been shown to bind to ELK1 (ETS-like gene 1), NFATc1 (nuclear factor of activated T cells 1) and LEF1 (lymphoid enhancer-binding factor 1) [116]. Moreover, when in complex with LMO2, the NFATc1 and LEF1 proteins exhibited increased and decreased transcriptional activity, respectively. LMO2 expression has also been reported in some B cell acute leukaemias (B-ALL) with differing prognostic correlations, where there is good prognostic association in some [115,118], but in others, such as B-ALL expressing the E2A–HLF fusion from t(17;19) (q22;p13), it is a poor prognostic feature [113,114].

Controversy surrounds the significance of LMO2 in epithelial cancers. One study reported LMO2 in 60% of pancreatic cancer samples and in 80–90% of high grade neoplasias tested, while being absent from normal pancreatic ductal epithelium [119]. However, tissue staining of a smaller set of pancreatic tumours did not find LMO2 expression except in the surrounding tumour vasculature [120], where it is known to be associated with tumour vascular remodelling [68]. The significance of LMO2 expression in pancreatic and other epithelial cancers requires more analysis. An intriguing observation has recently been published showing LMO2 exerting a stem cell phenotype in glioblastoma [84]. LMO2 could induce stem cell characteristics in mouse premalignant astrocytes and anti-LMO2 siRNA affected growth of human patient-derived glioma stem cells. Further studies are eagerly awaited.

## 12. Future of LMO2 therapy and LMO2 biology

While improved treatments have led to an increased survival rate, around one-fifth of paediatric T-ALL patients succumb to the disease, either through treatment resistance or relapse, while the figure for adult T-ALL is much higher [121,122]. *LMO2* suffers chromosomal translocation or activation by interstitial deletion in 5% of T-ALL and is aberrantly expressed in approximately 45% of T-ALL not exhibiting defects at the *LMO2* locus [33]. LMO2 is also expressed in the vasculature of many types of tumour [68,120], fulfilling its normal angiogenic role [67] in a disadvantageous setting. Increased angiogenesis is implicated in a number of clinical conditions, including eye diseases such as diabetic proliferative retinopathy, and also found to occur due to the inflammatory response in diseases such as rheumatoid arthritis and bowel disease (reviewed in [123]). Therefore, inhibition of LMO2 could be of clinical benefit for many cancers, but also for other diseases that have angiogenic processes.

In view of the role of LMO2 in multimeric protein complexes by PPI and its importance in several areas of haematopoiesis, development of LMO2 inhibitors is important but also very challenging, requiring new approaches and categories of molecule that will be efficacious and safe for use in the clinic. Relatively few small molecules that inhibit PPI have been developed, although the options are increasing (reviewed in [124]). At present, there are no small molecule inhibitors of LMO2. The protein is not an enzyme with an active site but rather works as part of the protein interaction network. The development of anti-LMO2 macrodrugs is under investigation as an alternative that will target the LMO2 PPI or the *LMO2* mRNA. The latter could be targeted for destruction through the use of small interfering (si)RNA molecules. In a manner analogous to micro (mi)RNA miR-223 [125], anti-LMO2 siRNA molecules could potentially prevent translation of *LMO2* mRNA. However, these small RNAs may produce off-target effects and are prone to rapid turnover within the cell. Targeting therapies to disrupt the function of the LMO2 protein may be more practicable. A peptide aptamer was developed that conferred a significant growth inhibition of transgenic LMO2 leukaemic cells in transplantation assays [50]. An alternative is the use of intracellular antibody fragments, in the form of a single-chain variable fragment and a single variable heavy chain (VH) that can readily be isolated using molecular biology techniques such as intracellular antibody capture (IAC; reviewed in [126]). The anti-LMO2 single VH domain developed in our laboratory through the IAC technology [100,127–130] binds to LMO2 with high affinity and induces a structural distortion, rendering LMO2 unable to efficiently nucleate the LMO2-complex [49] (figure 5*b*,*e*). Indeed, an exciting possibility is that these macrodrugs may be used to inform rational design of small molecule drug inhibitors of LMO2 with similar mechanistic properties.

Major problems with macrodrugs, as opposed to small molecule drugs, are cell penetration, immunogenicity, stoichiometry and target location inside the cell. Macrodrugs are generally too large to simply cross the cell membrane, and are at risk of degradation, natural clearing through the liver and possible immune response. Intracellular expression of the antibody fragment from nucleic acid would, however, ensure high levels of anti-LMO2 protein in target cells. As LMO2 has roles in the HSCs, erythropoiesis and angiogenesis, specific tumour targeting may be required. This could be achieved through immunoliposome or immune-nanoparticle delivery, using particles coated with antibody having recognition of a target cell surface antigen, for specific uptake. Another option is using therapeutic viruses engineered for specificity. Furthermore, enhancement of the anti-leukaemic effects could be achieved through linking effector domains to the intracellular antibody fragments, to accelerate LMO2 protein degradation or to initiate apoptosis of the tumour cells following LMO2 binding [131]. Ongoing work in our laboratory is being undertaken to answer these questions and evaluate ways to produce effective therapy for LMO2-dependent disease.

In the 25 years since its discovery, much has been learned about LMO2 and the other LIM-domain-only family members (reviewed in [19]). In addition to LMO2 in diseases like cancer, LMO2 has potential clinical benefit in the development of therapeutic stem cells, due to its ability to be involved in reprogramming differentiated cells to confer a stem cell-like phenotype [92,94] and in making autologous iHSC [93]. In all, LMO2 has proved to be a remarkable protein with diverse effects. Research into LMO2 and its functions continues, and we hope this will soon translate into better management of relevant human diseases.
